# Do People Regard Robots as Human-Like Social Partners? Evidence From Perspective-Taking in Spatial Descriptions

**DOI:** 10.3389/fpsyg.2020.578244

**Published:** 2021-02-05

**Authors:** Chengli Xiao, Liufei Xu, Yuqing Sui, Renlai Zhou

**Affiliations:** Department of Psychology, School of Social and Behavioral Sciences, Nanjing University, Nanjing, China

**Keywords:** human–robot interaction, spatial cognition, spatial descriptions, social cognition, perspective-taking, social skills

## Abstract

Spatial communications are essential to the survival and social interaction of human beings. In science fiction and the near future, robots are supposed to be able to understand spatial languages to collaborate and cooperate with humans. However, it remains unknown whether human speakers regard robots as human-like social partners. In this study, human speakers describe target locations to an imaginary human or robot addressee under various scenarios varying in relative speaker–addressee cognitive burden. Speakers made equivalent perspective choices to human and robot addressees, which consistently shifted according to the relative speaker–addressee cognitive burden. However, speakers’ perspective choice was only significantly correlated to their social skills when the addressees were humans but not robots. These results suggested that people generally assume robots and humans with equal capabilities in understanding spatial descriptions but do not regard robots as human-like social partners.

## Introduction

Since the word “robot” was conceived in a science fiction drama in 1920, many imaginative science fiction creators and aspiring roboticists have been trying to create robots that can collaborate and cooperate with humans. Especially, robots can understand humans’ spatial instructions to fetch objects, reach destinations, avoid obstacles, and so on (Lemaignan et al., 2017). Nevertheless, spatial interactions with others are not only spatial tasks but also social tasks, and people’s assumptions about others’ socialness influence their spatial behaviors toward others (Tversky and Hard, 2009; Zwickel, 2009; Shelton et al., 2012; Clements-Stephens et al., 2013; Tarampi et al., 2016; Gunalp et al., 2019). Therefore, studies on human–robot spatial interactions meet thorny issues: Do people regard robots as human-like social partners or just objects? And what are the mechanism and consequences of attributing socialness to robots? Investigating these questions may contribute to both robot development and human cognitive study (Broadbent, 2017; Hortensius and Cross, 2018).

In the current research, we addressed these issues by examining human speakers’ self-other perspective choice when describing locations to human and robot addressees. As social beings living in the three-dimensional world, humans often describe spatial locations to others to communicate, collaborate, and achieve other social interactions. People are very flexible, as they can describe spatial locations from either themselves or the addressee’s perspective. For example, when sitting at the dining table, a speaker can ask the person opposite to give her/him the spoon “on my left” or “on your right.” However, if the speaker is sitting opposite a service robot, whose perspective will the speaker choose to say? For both the speaker and the addressee, they can directly access the egocentric spatial relations through sensorimotor information from the body but have to do an extra mental transformation to convert the egocentric spatial relations to other-centric ones (e.g., Roßnagel, 2000; Schober, 2009; Galati et al., 2018). For the speaker, describing from self-perspective (e.g., “on my left”) is easier than from the addressee’s perspective (e.g., “on your right”) whereas for the addressee, understanding descriptions from the speaker’s perspective (e.g., the speaker’s description “on my left”) is more difficult than from self-perspective (e.g., the speaker’s description “on your right”). Therefore, under the same task context, the speaker’s same or different perspective choice for the human and robot addressees has been hypothesized as the evidence of whether people regard robots as human-like agents in spatial communication (Moratz et al., 2001; Tenbrink et al., 2002; Fischer, 2006; Carlson et al., 2014; Li et al., 2016; Zhao et al., 2016).

Based on the above hypothesis, several pioneer studies consistently revealed that human speakers treated robot and human addressees differently in spatial perspective-taking; however, the found differences were inconsistent across studies. Some earlier studies showed that speakers tended to take robots’ perspective when describing locations to robots. In contrast, recent studies found the opposite – speakers were more likely to take their own perspective when making spatial interactions with robots than with humans. For example, in earlier studies (Moratz et al., 2001; Tenbrink et al., 2002; Fischer, 2006), when verbally instructing either a dog-like pet robot, a metal insect, or a box-like robot to move to particular goal objects, participants exclusively described from robots’ perspectives. However, in recent studies, when instructing a robot or a human to find target objects on a table (Li et al., 2016) or in a house (Carlson et al., 2014), or when interpreting an ambiguous number (i.e., 6 or 9) (Zhao et al., 2016), participants were less likely to take the robots’ than humans’ perspectives.

Despite the inconsistency, the above studies generally agreed on the hypothesis that human speakers follow the principle of least collaborative effort (Clark and Wilkes-Gibbs, 1986) in perspective choices to both the human and robot addressees (Fischer, 2006; Carlson et al., 2014). According to this principle, the conversation partners, instead of minimizing the speaker’s or the addressee’s effort individually, take account of both sides’ effort and adapt their perspective to share the cognitive burden and facilitate their coordination. Specifically, speakers may shift their spatial perspectives according to various factors from themself, the addressees, and the environments (Duran et al., 2016; Pouliquen-Lardy et al., 2016; for a review, see Galati and Avraamides, 2013). For example, speakers may invest their effort to take the addressees’ perspective when speakers have high spatial abilities (Schober, 2009), when they believe the addressee is limited in spatial abilities or cannot provide feedback (Schober, 1993, 2009), or when they found the addressee’s perspective is easy to adopt (Tversky et al., 1999; Galati and Avraamides, 2015).

Therefore, the inconsistent findings among previous studies might attribute to their various inconsistencies in addressees, speakers, and tasks. First of all, the robot addressees are different across previous studies. Due to the rapid robotic development and widespread science fiction robot images (Złotowski et al., 2015), human speakers in earlier and recent studies may shift their assumption about robots’ capabilities from less to more capable than humans, resulting in more to less other-perspective choices for robot than human addressees. Moreover, the robots’ appearances are varied across previous studies (e.g., machine-like, animal-like, or human-like), which may induce different assumptions about robots’ capabilities (Krach et al., 2008; Takahashi et al., 2014; Zhu and Chang, 2020). Second, the human speakers are of different ages and from different countries. For example, German and United States participants were tested in earlier and recent studies, respectively; older adults were tested in Carlson et al. (2014). Therefore, the speakers may vary in many traits critical to human–robot interaction (Schweinberger et al., 2020), such as anthropomorphism tendency (i.e., attributing human-like characteristics to non-human entities; e.g., the wind has intentions) and spatial abilities, which was suggested to be related to people’s assumption about robots’ capabilities (Waytz et al., 2010; Severson and Lemm, 2016) and speakers’ perspective choice (Schober, 2009; Galati et al., 2019b). Lastly, the spatial description tasks are different among previous studies, which may induce different relative speaker–addressee cognitive burden (Pouliquen-Lardy et al., 2016) and shift speakers’ perspective choice differently to human and robot addressees. For example, across studies, the spatial scale was tabletop, room, or house; the number of distractor objects varied from 2 to 14; the addressees provided feedback or not.

Moreover, assuming robots are capable of doing spatial perspective-taking does not necessarily mean people regarding robots as social partners. Recent studies on visual–spatial perspective-taking (Shelton et al., 2012; Clements-Stephens et al., 2013) suggest that the interaction between social skills and perspective-taking performance rather than perspective-taking performance *per se* is the evidence of whether people regard the targets as social agents. In tasks similar to Piaget’s three-mountains perspective-taking test, Shelton and her colleagues (Shelton et al., 2012; Clements-Stephens et al., 2013) asked participants to judge photos taken from which targets’ perspective. The targets could be human-like dolls or objects such as triangles or cameras. The results showed that although people’s perspective-taking performances were equivalent across targets, their performances were correlated with social skills only when the targets were human-like dolls but not objects, suggesting people regarding human-like dolls but not objects as social agents. Since describing locations to other people is also a social task involving perspective-taking, speakers’ perspective choice may also be correlated with their social skills. Thus, measuring the correlation between speakers’ social skills and their perspective choices in spatial descriptions may reveal whether people regard robot addressees as human-like social beings.

Therefore, in this study, in order to further investigate whether human speakers regard robots as human-like addressees, we (1) examined human speakers’ perspective choice to human and robot addressees under various spatial description scenarios and (2) measured the correlations between human speakers’ social skills and their perspective choice to human and robot addressees.

**FIGURE 1:**
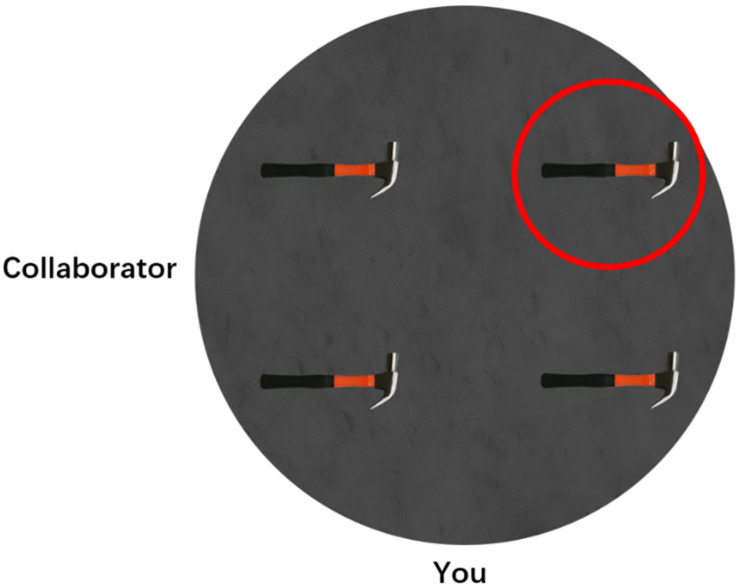
An example of experimental trials. The human addressee was labeled as “collaborator” and located at 270°, participant was labeled as “you” and located at 0°, and the target object was marked by a red circle.

The spatial description tasks were adapted from previous studies (Schober, 1993; Mainwaring et al., 2003); participants had to describe target locations to an imaginary robot or human addressee under scenarios varying in relative speaker–addressee cognitive burden. The human and robot addressees were presented as text labels (e.g., the human addressee was marked by the label text “collaborator” in Figure 1) and did not provide any feedback to speakers’ instructions. In this way, the interferences from addressees’ visual appearance and feedbacks were minimized. After the spatial description tasks, the speakers’ subjective concepts to their imaginary human or robot addressees were also measured. As there was no specific image or information of the robot and human addressees, the current study captures people’s general behaviors and concepts to typical humans and robots. The speakers were recruited from the same participant pool, and their critical individual differences, such as social skills, spatial abilities, and anthropomorphism tendency, were measured. Two environmental cues were simultaneously manipulated across trials to create spatial description scenarios varying in the relative speaker–addressee cognitive burden. The first cue was the relative difficulty of describing from the self- or other-perspective. Since the asymmetric spatial terms front/back are easier to produce and comprehend than the symmetric spatial terms left/right (Tversky et al., 1999; Mainwaring et al., 2003), therefore, we varied the symmetric and asymmetric spatial terms that could be used in self- and other-perspectives to create self-perspective easier, other-perspective easier, and equal difficulty trials^1^. The second environmental cue was the layout direction. Previous studies have found that both speakers and addressees tended to select the perspective aligned with the layout direction (Galati and Avraamides, 2015; Galati et al., 2019a). Therefore, in this study, the layout direction was varied by presenting non-directional objects, or directional objects aligning with the speaker or the addressee.

If under various spatial description scenarios, regardless of the relative speaker–addressee cognitive burden, speakers’ perspective choices were equivalent to human and robot addressees, it might suggest that speakers regard robot and human addressees of equal capabilities. Otherwise, if speakers made more self-perspective choices to one addressee than the other, it might suggest that they regard the former as more capable than the latter. Moreover, previous non-spatial human–robot speech interactions have shown that speakers might produce longer descriptions for robots than for human addressees, which suggested that participants put more linguistic effort into the description task for robots (Amalberti et al., 1993; Schmader and Horton, 2019). Therefore, we also examined the possible difference in description length and redundancy between human and robot addressee conditions. Further, the correlation between speakers’ social skills and their perspective choices might suggest whether they regard the spatial description task as social. Previous studies have shown that the speakers dominantly describe from the imaginary addressees’ perspective but decrease this other-centered tendency when tasks involving real social interaction, that is, when the partners are real rather than imagery (Schober, 1993; similar findings in addressees, Duran et al., 2011). Therefore, it was possible that in the current imaginary addressee task, speakers might also dominantly adopt addressees’ perspective, and speakers with higher social skills were more likely to assume the current imaginary addressees as real interactable social partners and took self-perspective to reduce self-cognitive burden.

## Materials and Methods

### Participants

Sixty-four university students (40 women), ages 18–29 years (*M* = 22.5, SD = 2.30), participated in return for monetary compensation. The ethics committee of psychology research of Nanjing University approved the study. Written informed consent was obtained from each participant before the experiments began. The number of participants was calculated by *a priori* Gpower 3.1 based on a pilot study data of 16 people (*Effect size* = 0.27, *Power* = 0.80).

### Materials and Procedures

All participants completed the following set of tests individually.

#### Describing Location Test

This test was adapted from previous spatial describing studies (Schober, 1993; Mainwaring et al., 2003). The test was programmed in JAVA and run on a laptop computer with 15.7-inch displays (resolution 1,920 × 1,080). Participants were informed that they were working with an imaginary partner and that their tasks were to describe the target object’s location to his/her partner. Half of the participants were informed that their partner was a human, and the other half were told that their partner was a robot. For each trial, participants watched a display on the laptop monitor. As shown in Figure 1, there were four identical objects within a circular area, and one of them was marked with a red circle as the target object. Participants were informed that their partners could not see the red circle. At the outer edge of the circular area, the participant’s position was marked by the label “you,” and the partner’s position was marked by the label “collaborator” or “robot” for the human or robot addressee, respectively. Participants were asked to speak out their spatial descriptions once they were ready, and their speeches were automatically recorded by the program. After finishing describing, participants pressed the space bar to initiate the next trial.

**FIGURE 2:**
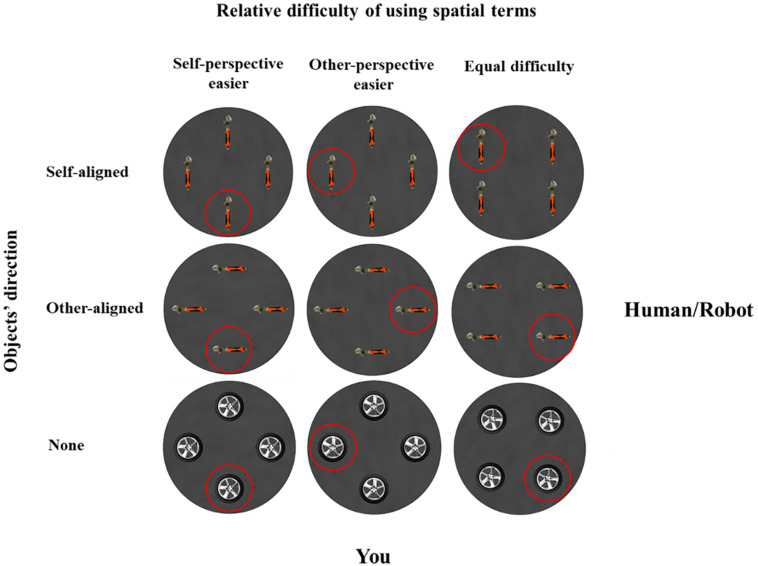
Examples of critical trials, organized as a function of the relative difficulty of using spatial terms (self-perspective easier, other-perspective easier, or equal) and object directions (aligned with self, other, or neither). The human or robot addressee was at 90°, and the participant was at 0°.

There were five practice trials and 80 experimental trials. Across trials, the participant always located at 0°, but the partner’s position was randomly presented at 0°, 90°, 180°, or 270°. In 48 critical trials, as shown in Figure 2, the partner’s location was presented at 90° or 270°. The layouts and the target objects were designed so that the relative difficulty of using spatial terms could be *self-perspective easier* (i.e., speakers can use asymmetric spatial terms front/back to describe from self-perspective but can only use symmetric terms left/right to describe from addressee’s perspective), *other-perspective easier* (i.e., use front/back from addressee’s perspective and left/right from self-perspective), or *equal* (i.e., equally use left/right and front/back from self and addressee’s perspective). Meanwhile, two directional objects (hammer and wrench) and two non-directional objects (wheel and gear) were used to create layouts so that the objects’ direction varied as aligned with the speaker, the addressee, or neither. To prevent participants from developing a fixed perspective selection strategy, we further varied the partner’s location by including 32 filler trials. In these filler trials, the partner’s location was presented at 0° or 180°; thus, the relative difficulty of using spatial terms was always equal between self- and other-perspectives. The objects were identical to the critical trials, and the objects’ directions were varied as in critical trials.

#### Concepts About the Addressee

After the spatial describing task, participants filled the Godspeed questionnaire (Bartneck et al., 2009) to measure their concepts of anthropomorphism, animacy, likeability, perceived intelligence, and perceived safety about their robot or human addressee. This questionnaire was initially designed to measure people’s concepts about the robot. In the current study, to compare participants’ concepts between human and robot addressees, and also as a manipulation check to confirm that the participants followed the instruction to imagine their addressees as a human or a robot, all the participants complete this questionnaire to evaluate their addressees. For each conception, participants had to rate their impression of the robot or human partner on 9-point semantic differential scales between two bipolar words, such as “mechanical–organic” or “unintelligent–intelligent.”

#### Speaker’s Individual Differences

At last, all the participants completed the Autism-spectrum Quotient scale (AQ; Baron-Cohen et al., 2001), Individual Differences in Anthropomorphism Questionnaire (IDAQ; higher score = higher anthropomorphism tendency; Waytz et al., 2010), and Perspective-Taking/Spatial Orientation Test (PTSOT; Hegarty and Waller, 2004) to measure their social skills, anthropomorphism tendency, and spatial abilities, respectively. Especially, for the social skills, we followed Shelton et al. to create a combined social/communication score by averaging the scores of social skill and communication scales in AQ, as the items in these two scales most closely aligned with typical social behaviors^2^. The original coding rules result “higher score = poorer social skills”; in order to reduce confusion in understanding the results, we reversed the rules to code social/communication score as “higher score = higher social skills.” In the PTSOT test, participants watched an array of objects and were asked to imagine standing at one object and facing the second object and then to point to the direction of the third object. The average absolute pointing errors across trials is the test score; therefore, a higher score means poorer spatial perspective transformation ability.

## Results

### Describing Location Test

The data in the describing location test were subject to 2 (Addressee: human vs. robot) × 3 (Relative difficulty: self-perspective easier vs. other-perspective easier vs. equal) × 3 (Layout direction: self-aligned vs. other-aligned vs. none) mixed-design ANOVA, with addressee as the between-participants variable.

#### Description Length and Redundancy

The description length (i.e., the number of Chinese characters in each description) was counted, the description redundancy (1 = redundant, and 0 = not redundant) was encoded in critical trials, and both were subjected to the mixed-design ANOVA. The redundancy was defined as that there were two or more ways to locate the target object according to the description; for example, there were both “on my left” and “on your right” in one description.

For the description length, only the main effect of relative difficulty was significant, *F*(2, 124) = 32.55, *p* < 0.001, $\eta_{p}^{2}$ = 0.34. Pairwise comparisons showed that the description lengths were shorter in the self-perspective easier condition (*M* = 9.51, SD = 0.32) than in the other-perspective easier (*M* = 11.22, SD = 0.44) and equal difficulty (*M* = 12.00, SD = 0.40) conditions, *F*(1, 62) = 27.13 and 78.59, *p*s < 0.001, $\eta_{p}^{2}$ = 0.30 and 0.56, respectively. The differences between the latter two conditions were also significant, *F*(1, 62) = 5.37, *p* < 0.05, η_*p*_^2^ = 0.08.

For the description redundancy, neither the main nor interaction effects were significant. The redundancy was low for both the human (*M* = 0.02, SD = 0.04) and robot (*M* = 0.03, SD = 0.04) addressee condition.

#### Perspective Choice

For critical trials in the describing location test, the perspectives of descriptions were encoded as 1 = addressee’s perspective (e.g., “The hammer on your left”), −1 = self-perspective (e.g., “The hammer on my left”), and 0 = both perspectives (e.g., “The hammer on your left and in front of me”). The overall average score was 0.73 (SD = 0.68), indicating that participants tended to describe from the addressees’ perspective in general.

**FIGURE 3:**
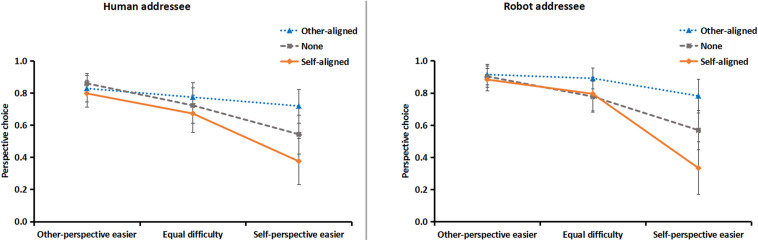
Results of perspective choice to human (left) and robot (right) addressees, as a function of layout direction and the relative difficulty of using spatial terms from self or other perspectives. 1 = addressee’s perspective, –1 = self-perspective, and 0 = both perspectives. Error bars represent the standard error of the mean.

As shown in Figure 3, the mixed-design ANOVA revealed four major findings:

First, participants’ perspective choices were equivalent for human and robot addressees, as neither the main effect nor any interaction of addressee was significant, *Fs* < 1, *p*s > 0.61.

Second, the main effect of relative difficulty was significant, *F*(2, 124) = 17.90, *p* < 0.0001, $\eta_{p}^{2}$ = 0.22. Compared with the equal difficulty condition, speakers were more likely to describe from self- or other-perspective when the spatial terms were easier from self- or other-perspective, *F*(1, 62) = 17.78 and 6.10, *p* < 0.0001 and 0.05, $\eta_{p}^{2}$ = 0.22 and 0.09, respectively.

Third, the main effect of layout direction was significant, *F*(2, 124) = 13.67, *p* < 0.0001, $\eta_{p}^{2}$ = 0.18. Compared with the none-orientation condition, speakers were more likely to describe from self- or other-perspective when the layout direction was aligned with self- or other-perspective, *F*(1, 62) = 15.51 and 7.38, *p* < 0.0001 and 0.01, $\eta_{p}^{2}$ = 0.20 and 0.11, respectively.

Fourth, the interaction between relative difficulty of spatial terms and layout direction was significant, *F*(4, 248) = 10.80, *p* < 0.0001, $\eta_{p}^{2}$ = 0.15. Planned contrasts showed that when spatial terms were easier for self-perspective, speakers were more likely to describe from self-perspective when objects orientation aligned with self than other, *F*(1, 62) = 20.11, *p* < 0.0001, $\eta_{p}^{2}$ = 0.25, whereas when spatial terms were easier for other-perspective, self- or other-aligned objects orientation did not shift speakers’ perspective choice, *F*(1, 62) = 1.01, *p* = 0.30.

#### Response Latency

**FIGURE 4:**
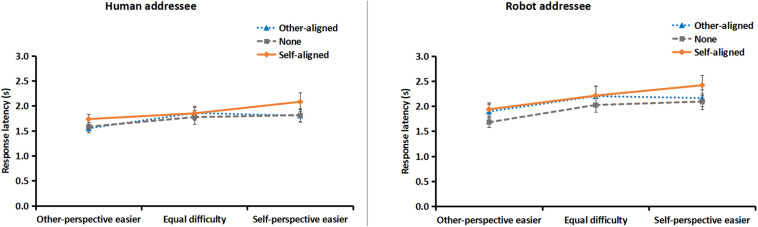
Results of response latency of descriptions from human (left) and robot (right) addressee’s perspective, as a function of layout direction and the relative difficulty of using spatial terms from self or other perspectives. Error bars represent the standard error of the mean.

To further reveal speakers’ cognitive burden when describing from addressees’ perspective, the response latencies of descriptions (i.e., from the presence of a scenario to the utterance of the first word) from the addressee’s perspective were computed for each participant under each condition. Data of 16 participants were not included in the further analysis because they did not describe from addressees’ perspective in one or more conditions. Therefore, response latencies from 48 participants (22 in robot and 26 in human addressee condition) were subjected to the mixed-design ANOVA and revealed four major findings (Figure 4).

First, whether the addressee was a human or a robot did not affect speakers’ response latency, as neither the main effect nor any interaction of addressee was significant, *Fs* < 1.60, *p*s > 0.21.

Second, the main effect of relative difficulty was significant, *F*(2, 92) = 6.69, *p* < 0.01, $\eta_{p}^{2}$ = 0.13. Speakers responded more quickly in other-perspective easier condition than in self-perspective easier and equal difficulty conditions, *F*(1, 46) = 9.44 and 8.50, *p*s < 0.01, $\eta_{p}^{2}$ = 0.17 and 0.16, respectively, suggesting the easier spatial terms (i.e., front/back) from addressee’s perspective facilitated producing descriptions from that perspective. The differences between the latter two conditions were not significant, *F*(1, 46) = 1.58, *p* = 0.22.

Third, the main effect of layout direction was significant, *F*(2, 92) = 8.52, *p* < 0.0001, $\eta_{p}^{2}$ = 0.16. Speakers responded more slowly in self-aligned condition than in other-aligned and none-orientation conditions, *F*(1, 46) = 7.00 and 22.14, *p* < 0.05 and 0.0001, $\eta_{p}^{2}$ = 0.13 and 0.33, respectively, suggesting the self-aligned layout direction impeded producing descriptions from addressee’s perspective. The differences between the latter two conditions was not significant, *F*(1, 46) < 1, *p* = 0.33.

Fourth, the interaction of relative difficulty of spatial terms and layout direction was significant, *F*(4, 184) = 2.46, *p* < 0.05, $\eta_{p}^{2}$ = 0.05. Planned contrasts showed that when spatial terms were easier for self-perspective, speakers responded more slowly when objects orientation aligned with self than other, *F*(1, 46) = 8.85, *p* < 0.01, $\eta_{p}^{2}$ = 0.16, whereas when spatial terms were easier for other-perspective, self- or other-aligned objects orientation did not affect speakers’ response latency, *F*(1, 46) = 1.58, *p* = 0.22.

### Concepts About the Addressee

Independent-samples *T* tests were employed to compare participants’ concepts with those of their human or robot addressee. Participants regarded robot addressees as less in anthropomorphism and animacy (*M* = 3.22 and 3.69, SD = 1.15 and 1.32) than human addressees (*M* = 5.28 and 5.35, SD = 2.21 and 2.55), *t*(62) = 4.68 and 3.28, *p*s < 0.005, confirming that they followed the instruction to imagine their addressees as a human or a robot. But they rated robot and human addressees in equal likeability, intelligence, and safety (for robot, *M* = 5.48, 5.69 and 5.60, SD = 1.14, 1.73, and 0.76; for human, *M* = 5.71, 6.01 and 5.81, SD = 1.56, 1.65, and 0.95), *t*s < 1, *p*s > 0.33.

However, participants’ subjective concepts to their human or robot addressees did not relate to their perspective choice, as none of the correlations was significant.

### Correlations Between Speakers’ Perspective Choice and Individual Differences

In order to examine the relationship between speaker’s perspective choice and their individual differences in social skills, anthropomorphism tendency, and spatial ability, data from 64 participants were split by the addressee (i.e., human or robot); and the correlations between perspective choice scores and social/communication, IDAQ, and PTSOT scores were computed. As shown in Table 1, there were three major findings:

**TABLE 1:** Correlations between perspective-choice scores and the social/communication, anthropomorphism (IDAQ), and Perspective-Taking/Spatial Orientation Test (PTSOT) scores under human or robot addressee conditions.

Perspective choice		Social/Communication		IDAQ		PTSOT	
		Human	Robot	Human	Robot	Human	Robot
Equal difficulty	None	−0.609**	0.252	–0.162	−0.407*	0.149	0.008
	Self-aligned	−0.411*	0.250	–0.057	−0.432*	0.167	0.067
	Other-aligned	−0.442*	0.002	0.041	−0.482*	0.139	0.029
Self-perspective easier	None	−0.431*	0.212	–0.244	–0.191	0.211	–0.060
	Self-aligned	−0.429*	0.298	–0.253	–0.015	0.233	–0.097
	Other-aligned	−0.384*	0.030	0.024	–0.253	0.222	–0.230
Other-perspective easier	None	−0.418*	0.247	0.070	−0.628**	0.110	0.097
	Self-aligned	−0.485**	0.262	0.111	−0.595**	0.122	0.103
	Other-aligned	−0.377*	0.091	–0.009	−0.625**	0.140	0.102
Average across conditions		−0.510**	0.239	–0.089	−0.415*	0.200	–0.025

*p < 0.05; **p < 0.01.

**FIGURE 5:**
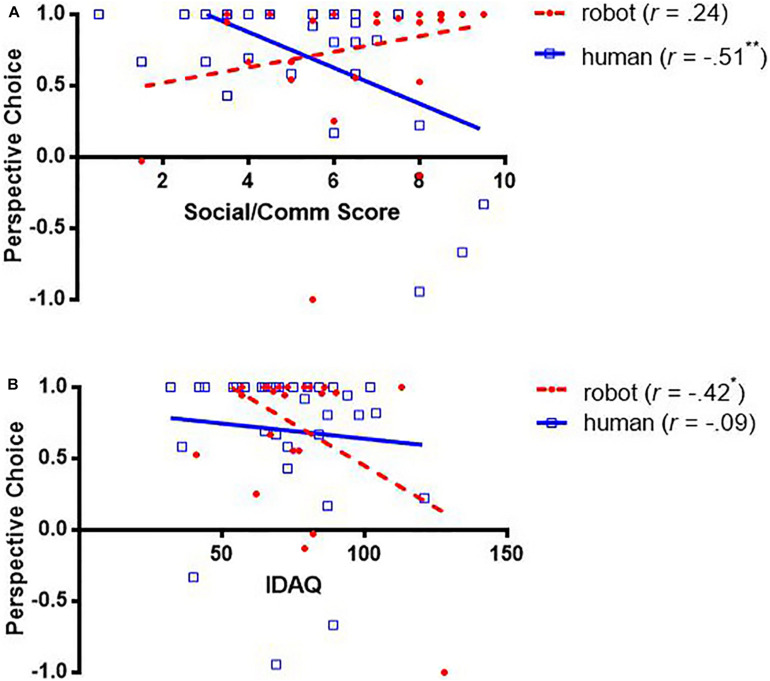
Correlations between perspective choice and social/communication scores **(A)** and Individual Differences in Anthropomorphism Questionnaire (IDAQ) scores **(B)**.

First, speakers’ social skills were only significantly associated with their perspective choice when the addressees were humans (*r*s > 0.384, *p*s < 0.05) but not robots, suggesting they only regard humans but not robots as social partners. As predicted, speakers with higher social skills (i.e., higher social/communication scores) were less likely to describe from other-perspective (i.e., lower perspective-choice scores) (Figure 5A).

Second, speakers’ anthropomorphic tendencies were associated with their perspective choice only when the addressees were robots but not humans. Speakers with higher anthropomorphic tendencies (i.e., higher IDAQ scores) were less likely to describe from the robots’ perspective (i.e., lower perspective-choice scores) (Figure 5B).

Third, speakers’ spatial perspective transformation ability was not significantly associated with their perspective choice.

## Discussion

In this study, we investigated whether human speakers regarded robots as human-like addressees by two means. On the one hand, following the traditional approach, under various spatial scenarios, we observed that speakers’ perspective choices to imaginary human and robot addressees were equivalent regardless of diverse relative speaker–addressee cognitive burden. On the other hand, adapted from studies in visual–spatial perspective-taking (Shelton et al., 2012; Clements-Stephens et al., 2013), we measured the correlation between speakers’ social skills and perspective choice, and we found that it was only significant when the addressees were humans but not robots. The above results suggested that although human speakers described spatial locations in similar ways to imaginary human and robot addressees, they only regarded humans but not robots as social partners.

Unlike previous studies, in which speakers were more or less likely to describe from the robot than the human addressees’ perspective, in this study, speakers made similar perspective choices for their imaginary robot and human addressees regardless of various relative speaker–addressee cognitive burden. According to the principle of least collaborative effort, these results suggested that our speakers assume their robot and human addressees of equal capabilities. In the current study, text labels rather than specific images were used to present human and robot addressees, and none of them provided feedback. These experimental settings allowed us to capture people’s general expectations of humans’ and robots’ capabilities and socialness. Because both top-down (e.g., introduction about robots’ capabilities and socialness; Stenzel et al., 2012; Vollmer et al., 2015; Cross et al., 2016; Baker et al., 2018) and bottom-up cues (e.g., robots’ appearance, voice, or behavior; Fischer, 2006; Krach et al., 2008; Eyssel and Hegel, 2012; Waytz et al., 2014) can shift people’s assumption and behavior toward robots, future studies with varying top-down and bottom-up cues may deepen our understanding about it.

Moreover, the current task was relatively simple, as participants only need to describe locations in a four-object regular layout rather than describing routes or locations in complicated environments or interactively communicating with others. Therefore, further studies are needed to verify the current findings across culture and in more difficult or interactive tasks, which may tax more spatial and social abilities and measuring more speech index such as speech entrainment (Branigan et al., 2011; Beňuš, 2014), repair of misunderstandings (Corti and Gillespie, 2016), referents expressions and conceptualizations (Schmader and Horton, 2019), and dynamic changes over time (Schober, 2009; Dale et al., 2018).

In the current study, two environmental cues were simultaneously manipulated to vary the relative speaker–addressee cognitive burden, which both significantly affected speakers’ perspective choice and response latency. When spatial terms were easier for self- than other-perspective, and when the layout direction was aligned with self than other’s perspective, speakers were less inclined to describe from addressees’ perspective; if they kept on describing from the addressees’ perspective, their response latencies significantly increased, indicating they had to invest more effort to produce spatial descriptions. Moreover, the interactions between the two environmental cues indicated that the cues’ effects were additive on perspective choice. When both cues suggested self-perspective (i.e., spatial terms were easier in self-perspective, and layout direction was self-aligned), speakers were least inclined to describe from the addressees’ perspective. However, only the relative difficulty of spatial terms but not the layout direction affected description length, suggesting that the two environmental cues have different effects on speakers’ linguistic effort. Together, the above findings suggested that different speech indexes (perspective choice and response latency vs. description length) might reveal different aspects of the speaker’s cognition, and the two environmental cues might affect speech production in different ways.

The similar perspective choice to human and robot addressees is not likely due to participants failing to follow the instruction to imagine their addressees as humans or robots. First, such instruction manipulations about conversation partners have been proved to be effective in previous studies (Duran et al., 2011), in which participants successfully followed instructions to conceive their speakers as imaginary or real. Second, in this study, participants rated robot addressees less in anthropomorphism and animacy than human addressees, confirming that they did imagine their addressees as humans or robots. Moreover, on the other hand, these findings suggested that robot addressees’ anthropomorphism and animacy may not be related to speakers’ perspective choice, as speakers made similar perspective choices to human and robot addressees even though they assumed robots less in anthropomorphism and animacy than human.

Although speakers made similar perspective choices to human and robot addressees, their individual differences in social skills and anthropomorphism tendency were differently correlated with perspective choices to human and robot addressees. The correlation between speakers’ perspective choice and social skills were only significant when the addressees were humans but not robots, suggesting human speakers take their human but not robot addressees as social partners. The correlation between speakers’ perspective choices and anthropomorphism tendency was only significant when the addressees were robots but not humans, which further confirmed that the participants assume robots differently from humans. Previous studies have shown that people with higher anthropomorphism tendencies are inclined to regard robots of higher capabilities (Waytz et al., 2010; Severson and Lemm, 2016). Therefore, in this study, speakers with higher anthropomorphism tendencies may also be inclined to regard their robot addressees of higher capabilities in understanding spatial instructions and then following the principle of least collaborative effort to describe more from self-perspective to reduce self-cognitive burden (Schober, 2009). Inconsistent with previous studies (Galati et al., 2019b), the current study did not find significant correlations between speakers’ spatial perspective transformation abilities and perspective choices, which might be due to the current relatively simple task that do not require high spatial perspective transformation abilities. In the current study, participants only need to describe a target location among four candidate objects, whereas in Galati et al. (2019b), participants have to describe and understand a complex route, which involved multiple spatial perspective transformation and tracking.

As hypothesized, when the addressees were humans, speakers with higher social skills were more likely to take self-perspective, which might be explained as they assumed the current imaginary addressees as real interactable social partners and took self-perspective to reduce self-cognitive burden. Similar to previous non-feedback situations (Schober, 1993), speakers in this study dominantly described from addressees’ perspective. This preference is explained as the speaker tried to minimize their collaborative effort (Clark and Wilkes-Gibbs, 1986). However, as there was no feedback from the addressees, the speakers could not specify the extent to which they mostly minimized the collaborative effort. Under this imaginary context, people’s social skills might help them make a sound estimation and adjust their perspective choice. It is possible that speakers overly take the cognitive burden from their imaginary addressee by overly adopting addressee-centered descriptions, and speakers with higher social skills might more properly against this unnecessary tendency and shift to more egocentric perspective choices, as they are communicating with real interactable addressees. Moreover, it is also possible that people with lower social skills encounter more social rejections in everyday life, which may increase their spatial perspective-taking behavior when interacting with others (Knowles, 2014), regardless of whether they are real or imaginary.

For those speakers with poorer social skills, they were more inclined to take the addressees perspective, which seems to conflict with the finding that people with poorer social skills had difficulty in taking other’s visual–spatial perspectives (Shelton et al., 2012; Clements-Stephens et al., 2013) and behave in a less sociable way (Baron-Cohen et al., 2001). For the visual–spatial perspective-taking, it worth to note that, first, people with poor social skills performed visual–spatial perspective-taking quite well, as their accuracies were mostly over 50%, much higher than the random rate of 14.29% (one out of seven targets); second, in current tasks, the spatial scenarios were very simple, and there was no time pressure for speakers to generate their instruction; the absence of correlations between perspective choices and PTSOT confirmed that speakers’ perspective choice was not related to their visual–spatial perspective-taking abilities.

Although people did not regard robots as social partners in the current study, they regarded wooden human models and fashion dolls as social agents in visual–spatial perspective-taking tasks, as their performances were related to their social skills (Shelton et al., 2012; Clements-Stephens et al., 2013). This discrepancy might due to people require more or less socialness from the agents to regard them socially in different tasks. In the visual–spatial perspective-taking task, a social agent only needs a little animacy (e.g., can see the scene), which could be inferred from the human-like appearance of models and dolls, whereas in the current spatial communication task, people might require more human-like traits from their addressees before they can regard them as social partners, because social addressees not only can see the scene but also can understand the verbal instruction and match it with scenes from multiple perspectives. The agent’s socialness is a continuum constructed across multiple dimensions and influenced by both bottom-up and top-down cues (Fischer, 2011; Hortensius and Cross, 2018). In the current study, as the human and robot addressees were presented in text labels, speakers attribute their socialness based on top-down rather than bottom-up cues, whereas in the robotics community, there are kinds of robots that vary in forms, shapes, and types. Further studies on the specific effects of various bottom-up and top-down cues on the attribution of socialness to robots might contribute to the robotic design and understanding of the agent’s socialness. Besides, speakers’ general expectations of humans and robots (Evers et al., 2008) and concepts of agency (Ojalehto et al., 2017) might vary across cultures; therefore, cross-cultural studies could also contribute to the understanding of this area.

In summary, by asking people to make spatial description and to rate the imaginary human or robot addressees, the current study suggested that on the one hand, people regarded robots as of equal capabilities to humans in understanding spatial descriptions, as they made similar perspective choices to human and robot addressees regardless of the various relative speaker–addressee cognitive burden, while on the other hand, people only regarded human but not robot addressees as social partners, as their social skills only related to their perspective choice when the addressees were humans. In other words, spatial communication is both spatial and social tasks, and in human speakers’ general expectations, robots are of human-like spatial capabilities but not human-like social partners. These findings further reveal people’s behavior and concepts toward robots, provide insights into the nature of social agents, and suggest examining the interaction with social skills is a novel effective way to investigate whether people regard robots as social partners. Moreover, the current research raises the old but important issue – what is the ultimate goal in robotics? It is clear that we want robots to become more capable, in language communication and other domains. However, is it necessary to build robots as social as humans? Investigating this issue may help us better understand human and build better robots that fit humans’ needs.

## Data Availability Statement

The raw data supporting the conclusions of this article will be made available by the authors, without undue reservation.

## Ethics Statement

The studies involving human participants were reviewed and approved by the Ethics Committee of Psychology Research of the Nanjing University. The patients/participants provided their written informed consent to participate in this study.

## Author Contributions

CX and RZ developed the study idea and designed the experiment. LX implemented the study and performed the data collection. CX, LX, and YS contributed to the data analyses and manuscript drafting. All authors contributed to the article and approved the submitted version.

## Conflict of Interest

The authors declare that the research was conducted in the absence of any commercial or financial relationships that could be construed as a potential conflict of interest.

## Footnotes

1 In Chinese, the linguistic complexities were equal between front/back and left/right. Chinese speakers use the same sentence structure “on my/your/it left/right/front/back” (在我的/你的/它的左边/右边/前面/后面, *zai wo-de/ni-de/ta-de zuo-bian/you-bian/qian-mian/hou-mian*) to describe each location. As English speakers, Chinese speakers also use the body axes to interpret egocentric direction words and respond to spatial terms front/back faster than to right/left (Mou et al., 2004).

2 The other three scales in AQ measure an individual’s perseveration, attention to detail, and imagination, which are associated with autism spectrum disorders but little related to social behaviors.
